# Size matters: How reaching and vergence movements are influenced by the familiar size of stereoscopically presented objects

**DOI:** 10.1371/journal.pone.0225311

**Published:** 2019-11-20

**Authors:** Rebekka S. Schubert, Maarten L. Jung, Jens R. Helmert, Boris M. Velichkovsky, Sebastian Pannasch

**Affiliations:** 1 Faculty of Psychology, Technische Universität Dresden, Dresden, Germany; 2 National Research Center “Kurchatov Institute”, Moscow, Russian Federation; 3 Moscow Institute for Physics and Technology, Moscow, Russian Federation; 4 Russian State University for the Humanities, Moscow, Russian Federation; University of Exeter, UNITED KINGDOM

## Abstract

The knowledge about the usual size of objects—familiar size—is known to be a taken into account for distance perception. The influence of familiar size on action programming is less clear and has not yet been tested with regard to vergence eye movements. In two experiments, we stereoscopically presented everyday objects, such as a credit card or a package of paper tissues, and varied the distance as specified by binocular disparity and the distance as specified by familiar size. Participants had to fixate the shown object and subsequently reach towards it either with open or with closed eyes. When binocular disparity and familiar size were in conflict, reaching movements revealed a combination of the two depth cues with individually different weights. The influence of familiar size was larger when no visual feedback was available during the reaching movement. Vergence movements closely followed binocular disparity and were largely unaffected by familiar size. In sum, the results suggest that in this experimental setting familiar size is taken into account for programming and executing reaching movements while vergence movements are primarily based on binocular disparity.

## Introduction

### Familiar size as a cue to depth perception

The size of the retinal image created by an object is inversely proportional to its distance from the observer. If the object and therefore its size is familiar to the observer, the distance of the object can be calculated from the retinal image. For example, when we see a car and the car appears quite small, we conclude that the car is far away. This pictorial depth cue is called familiar size. Most objects we encounter are familiar to us, if not as an individual then as belonging to a category of objects. For example, we might not have seen the car before but we know how big cars typically are.

Familiar size is important for relative depth perception. For example, when pictures of a golf ball and a baseball with the same size are presented, the golf ball appears closer [1]. Further, familiar size is relevant for judgements of perceived depth for exocentric as well as egocentric distances. For example, judgements of the depth between a dollar bill and an abstract disk (exocentric distance) were different when the dollar bill was a smaller reproduction compared to one with the correct size [2]. Verbally expressed distances to familiar objects (egocentric distance) were also influenced by familiar size [3]. Generally, studies revealed that the familiar size of an object influences depth perception especially when depth cues were reduced and binocular disparity was not available [4]. To sum up, there is evidence that familiar size can affect conscious depth perception. However, according to the influential theory of the two visual systems there are two separate pathways for perception and action [5, 6]. Well-learned actions like reaching and grasping are assumed to be controlled primarily by bottom-up information independent of object recognition and top-down object knowledge. Therefore, the question remains:

### Does familiar size influence reaching movements?

There is a number of studies on familiar size regarding grasping, yet only few concerning reaching movements. First, we will sum up the studies concerning grasping.

By directly investigating the use of familiar size for movement planning, Marotta and Goodale [7] found no differences in grasping movements when binocular disparity was available and familiar size was added as additional depth cue. They concluded that under normal viewing conditions binocular cues dominate visuomotor control. The work was criticized by McIntosh and Lashley [8]) due to the use of featureless spheres the size of which was learned during the experiment These authors used matchboxes of common brands instead and found that familiar size influenced grasping movements even when binocular disparity was present. Similarly, Borchers and Himmelbach [9] reported a better grasp scaling for familiar everyday objects with known sizes compared to meaningless cuboids for grasp movements with and without visual feedback. They conclude that the familiar size of objects encoded in long-term memory is exploited for planning and controlling grasping movements even under unconstrained, binocular viewing conditions. However, in these studies the objects are actually grasped and thus both visual and haptic feedback is provided. Since the experimental design allows participants to learn associations between specific objects and related grasping movements, one cannot rule out the possibility that the effect might be more a learned association and less an effect of familiar size. Indeed, it has been shown that under certain conditions participants are able to learn such associations for unfamiliar objects, e.g. when objects are distinct, when objects are fully textured, and when only one or few distances are used [10–15]. Thus, results on grasping with haptic feedback are not transferable to reaching movements without feedback.

In a series of experiments, Sousa and colleagues [16–18] presented unfamiliar objects (unicolored cubes) stereoscopically and asked participants to reach to the location of the objects without visual or haptic feedback. When the size of the object did not vary, participants formed assumptions about the size of the object mainly based on the size of the object in the previous trial. Those assumptions about the size of the object were taken into account by the participants for their reaching movement. Therefore, it seems that we generate assumptions about likely sizes of objects and use those assumptions to plan reaching movements without visual feedback. However, these findings do not allow conclusions about the influence of long-term familiarity with the size of familiar objects.

In order to reduce the likelihood of being unfamiliar with the shown object, we stereoscopically presented an everyday object, namely a tennis ball supposing that its size was familiar to all the participants [19]. To our knowledge, this was the first study to test the influence of familiar size on reaching estimates. We asked participants to either perform the reaching movement with eyes closed and thus without feedback or with eyes open and thus with visual (but no haptic) feedback. We found that depth information from binocular disparity and from familiar size was combined and that the effect of familiar size was larger when no visual feedback was available. This difference between feedback conditions was consistent with other findings showing differences between so called vision-guided and memory-guided reaching [20, 21].

However, since we presented the same tennis ball in all trials, we cannot generalize the results to other objects. Furthermore, using only one object might have led to an over- or underestimation of the true effect of familiar size. An overestimation is possible since Sousa and colleagues [16] showed that participants gave more weight to size as a cue for distance when the size of the presented objects varied only slightly. An underestimation is possible since most of the participants of the study reported that they do not interact with tennis balls on a regular basis. Thus, the size of the tennis ball might not have been as familiar to them as the size of objects with which they interact on a daily basis as, for example, a package of paper tissues.

The aim of the current study is to test whether the effect of familiar size on reaching movements with and without visual feedback can be replicated with other objects. Therefore, we stereoscopically presented six different objects and varied the distance as specified by binocular disparity and the size of the familiar object.

So far, we have discussed binocular disparity and (familiar) size as important depth cues for grasping and reaching. However, in personal space—the area surrounding the observer within arm’s reach and slightly beyond—vergence is another relevant depth cue [22]. An influence of familiar size on vergence movements could also be possible and contribute to the effect of familiar size on reaching movements. Therefore, our second research question is:

### Does familiar size influence vergence movements?

Vergence movements are thought to be mainly driven by binocular disparity. Thus, most research on vergence movements has been done using either disparity step stimuli or disparity ramp stimuli, which means that an abstract object like a cross or a vertical line changes disparity either abruptly or smoothly. Consequently, most models on the control of vergence movements are based on those experiments and use disparity as the input signal [23, 24]. However, vergence can be influenced by other depth cues as well. For example, faster and more accurate vergence movements were obtained when additional depth cues were added to binocular disparity [25, 26]. Furthermore, in monocular viewing—when there is no binocular disparity—vergence movements can be induced by perspective line drawings [27], motion parallax [28], or the kinetic depth effect, i.e. the perception of three-dimensional structure from two-dimensional images changing over time [29].

In the studies mentioned above, binocular disparity was not available or it was congruent with monocular depth cues. There is ambiguous evidence for the influence on vergence movements when monocular depth cues or illusive perceptions are in conflict with binocular disparity. On the one hand, there are studies showing that vergence follows binocular disparity [30–32]. For example, using random-dot patterns González and colleagues [30] showed that when binocular disparity was in conflict with looming—perceived motion in depth due to the rapid change of object size—conscious perception followed looming, but vergence movements followed binocular disparity. On the other hand, vergence has been found to be influenced by monocular depth cues or illusive perceptions, at least to some extent [28, 33–38]. For example, when an observer looks at the nose of a hollow mask, binocular disparity would specify that the nose is farther away than the rest of the mask while the observer often illusively perceives a normal convex face with the nose being closer to him than the rest of the mask. In the case of this hollow mask illusion, vergence movements followed the illusion irrespective of whether a real mask [34] or a virtual mask [35] was presented. Compared to trials showing a convex face, vergence movements had longer latencies and smaller amplitudes in the illusion trials [35]. Thus, under certain conditions other depth cues or illusive perceptions are taken into account for the programming of vergence movements even when binocular disparity is available.

However, to our knowledge, so far it has not been examined whether the depth cue familiar size is used for the control of vergence movements. To investigate this question, we measured vergence movements in addition to reaching movements. The overall aim was to investigate whether familiar size is considered for controlling reaching and vergence movements.

## Experiment 1

### Methods

To test the influence of familiar size on distance perception in personal space, we systematically varied the size of familiar everyday objects while measuring vergence eye movements and reaching movements. As results might depend on whether or not visual feedback is available during the reaching movement, we instructed participants to reach with eyes open (sighted reaches) or with eyes closed (blind reaches).

#### Participants

Data from 26 participants were obtained. All participants were right-handed as indicated by the Edinburgh Handedness Inventory [39]. Visual acuity was tested binocularly with the Freiburg Visual Acuity Test [40] in 90 cm viewing distance. To be included, participants had to achieve a visual acuity of at least 1.0, which corresponds to a vision of 20/20. Stereo acuity was tested with Section B of the Random Dot Stereo Acuity Test (Vision Assessment Corporation). Only participants with a stereo acuity of 40‘‘ or lower were included. All participants passed a color vision test [41]. Although meeting all inclusion criteria, one participant had to be excluded from further analyses as his vergence movements did not follow the distance of the presented objects. Thus, all reported analyses are based on the remaining 25 participants: 14 women and 11 men aged between 19 and 40 years (*M* = 22.84, *SD* = 4.83). Participants were naive to the purpose of the study. Written informed consent was obtained prior to the study. Participants were compensated for their participation with either € 16 or course credit. The experimental procedure was approved by the local ethics committee (vote EK 315082014 of the ethics committee of the Technische Universität Dresden, Germany).

#### Experimental setup

Participants were seated individually in a dimly lit room. The experiment was presented on a Mitsubishi WD-60737 screen with a refresh rate of 60 Hz. This display is a rear-projection DLP^™^ TV. Since the whole screen exceeded the visual field, we used a screen resolution of 1064 by 598 pixels on a visible screen size of approximately 77 by 43 cm. Participants were seated 90 cm in front of the display with the eyes in the height of the center of the screen; the resulting viewing angle was approximately 46° horizontally and 22° vertically. However the stimuli were presented in the center of the screen. For image separation, participants wore shutter glasses (3D Vision glasses by Nvidia Corporation, Santa Clara, USA) which were synchronized with the display.

To control the experiment, participants used a Cedrus Response Pad RB-840 (cedrus, San Pedro, USA). The response pad was placed on the table in front of the participant with the button for the right index finger 65 cm in front of the display and 48 cm below the middle of the display where the objects were presented.

Eye movements were recorded binocularly with a sample rate of 500 Hz using the SR Research Ltd. EyeLink 1000 Plus eye-tracking system (SR Research, Ontario, Canada), with a spatial resolution below 0.01° and a spatial accuracy of better than 0.5°. Head movements were restrained by a chin and forehead rest.

**Fig 1:**
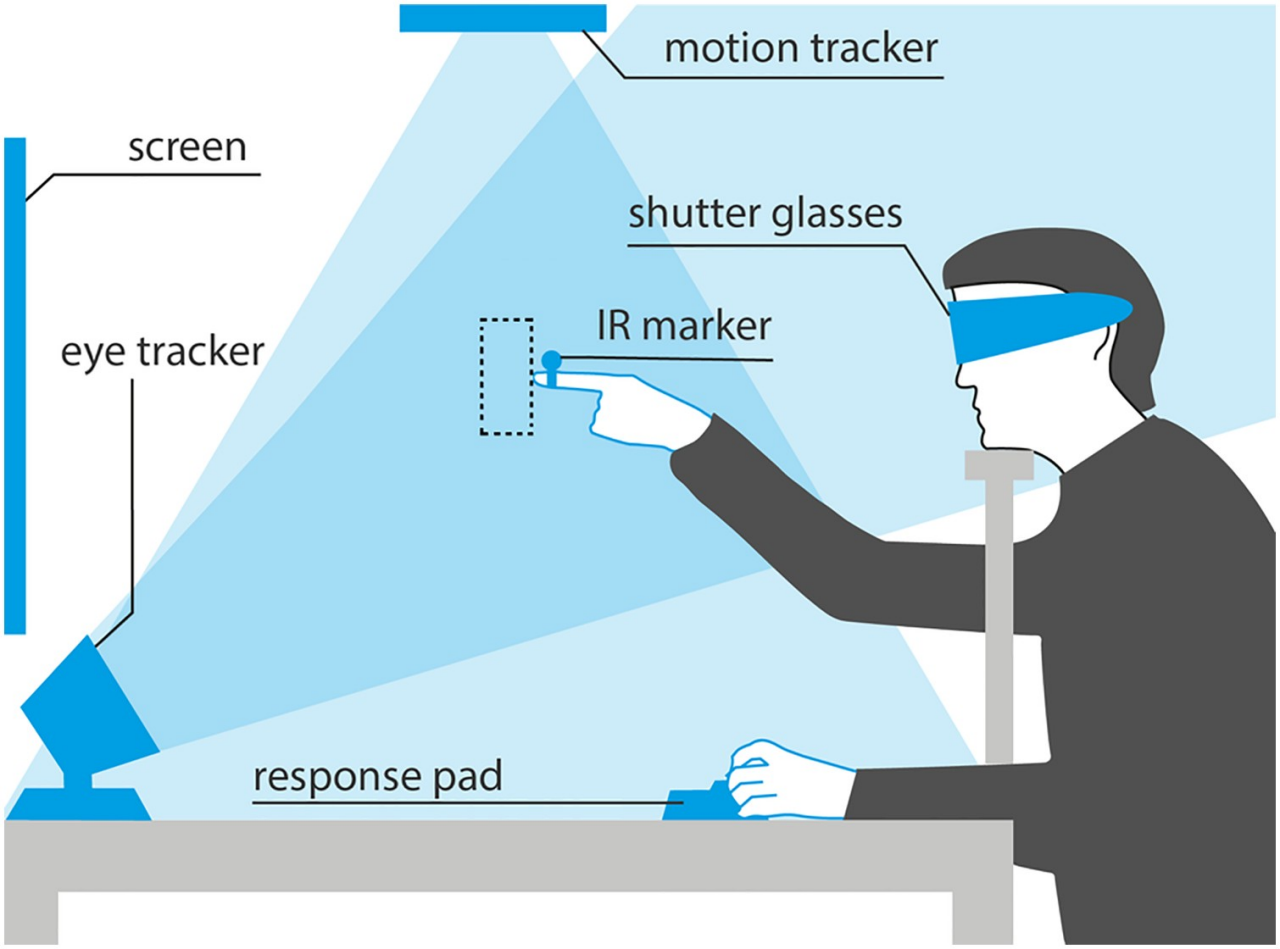
Experimental setup. Schematic representation of the experimental setup (not drawn to scale, forehead rest not shown); adapted from [19, 43, 44].

Reaching estimates were recorded using the marker-based optical tracking system OptiTrack V120: TRIO (NaturalPoint, Inc.), which was mounted above the participants’ seat. It consists of three 640 * 480 VGA sensors, has a capture speed of 120 FPS and—according to the manufacturer—sub-millimeter accuracy. A reflective sphere with a diameter of 9.5 mm was attached to the right index finger. The distance from the tip of the finger to the marker was measured individually. The position of the marker was streamed via the NatNet SDK to Vizard 5.8 (WorldViz, Santa Barbara, USA), a Python based VR development platform. Vizard was also used for the creation and rendering of the objects, for handling the experimental flow and recording experimental data. Rendering was done using participants’ individual interpupillary distances. These were measured with a pupilometer (Essilor PRC), which in an earlier study had an interrater reliability of *r* = .74 and a retest reliability of *r* = .94 [42]. A schematic representation of the experimental setup can be seen in Fig 1.

#### Stimuli

The stimuli in the experiment consisted of six different everyday objects. The six objects were selected after a pilot study with 20 participants (11 female) between 18 and 41 years old (*M* = 29.45, *SD* = 5.01). Pictures of 15 everyday objects with normed or standardized size were shown to the participants. Participants were asked to show the height and width of the presented object with their fingers and distance between the fingers was measured. The five objects with the smallest deviation of the size estimates from the real sizes were selected for the experiment: A five Euro note, a credit card, an empty toilet paper roll, a 250 g package of butter from a well-known local brand, and a package of paper tissues from a well-known brand. Additionally, the tennis ball was selected for comparability with our earlier experiments [19, 43]. For the selected objects, textured 3D models were bought (www.turbosquid.com) or created within Vizard using scans as textures. Great care was taken to ensure that the size of the virtual objects corresponded to the size of the real objects.

#### Procedure

Once the informed consent was given, the vision tests were run, and a demographic questionnaire was completed using LimeSurvey with a local server [45]. Next, participants were seated on a chair facing the display with the position of the eyes carefully adjusted with the help of the position tracking system.

The experiment was divided into two blocks, one for sighted and one for blind reaches. The order of the blocks was balanced across participants. Before each block the eye-tracker was calibrated using a standard nine-point binocular calibration, followed by a nine-point validation procedure. Each block consisted of 6 trials for exercise, which were not analyzed, followed by a new calibration and validation and 42 experimental trials. During the exercise trials, the objects had the correct size and were shown with a distance of either 25, 35, or 45 cm from the participant’s eyes. Each object was presented once, whereby the distance-object combination varied randomly for each participant. In the experimental trials the objects were presented in the distance as specified by disparity of either 30 or 40 cm. Each object was presented once at 30 and once at 40 cm in the correct size. To test the influence of familiar size, each object was also presented in the distance of 30 cm with a smaller size suggesting a distance of 40 cm and in the distance of 40 cm with a larger size suggesting a distance of 30 cm. Additionally, to prevent participants from remembering the distances and to reduce the rate of trials in which there was a conflict between disparity-specified distance and familiar size-specified distance, each object was presented at the distance of 25, 35, and 45 cm once in the correct size. Thus, in 28.6% of the experimental trials, there was a conflict of the distance specified by binocular disparity or specified by familiar size. Objects always appeared in the center of the screen at eye height with the side facing the participant at the intended distance.

The procedure of one trial was as follows: After a drift correction with the stimulus (a grey ring) on the level of the screen (with zero parallax), the object was presented. Participants were allowed to look at the object for unlimited time. When they felt they had a good image of the object and its position, they looked in the center of the object and pressed a button with the right index finger. 1000 ms after the button press, there was an acoustic signal telling the participants that they could start the reaching movement. In blind reaching trials, the object was blanked out with the acoustic signal and participants closed their eyes to prevent visual feedback. In sighted reaching trials the object and the fingers were visible throughout the whole reaching movement. Participants performed the reach with their right index finger outstretched to the position where they would touch the surface of the object with their finger tip. Participants were instructed to make a “fast and natural” movement. The participants terminated their reach by pressing the button with their left index finger. Subsequently, they laid their right hand back on the response pad and for blind reaches opened their eyes again. Next, a text message was displayed on the level of the screen, asking the participants whether they saw a 3D image or a double image. Participants answered by pressing with their left or right middle finger, respectively, and confirmed their response via the same button. Then participants started the next trial with a button press of the right index finger.

#### Data preparation

Data preparation and analyses were done using SPSS 20 [46], R 3.5.0 [47] and Gaze3DFix [48]. Raw data from the eye-tracking system and from the motion tracking system were processed to obtain the following five dependent variables: Vergence distance, vergence latency, maximal vergence velocity, reaching distance, and reaching duration.

Raw data from the eye-tracking system were transformed from pixel to mm and to a coordinate system with the origin at the center of the screen. All samples corresponding to blinks were eliminated and replaced via interpolation by 3rd order splines using the R package zoo [49]. The first 50 ms of each trial, when participants looked at the position of the drift correction ring, were defined as the baseline. For this period, we calculated the median of the positions of the left and the right gaze, respectively. We used these as an individual baseline for each trial and subtracted the values from the gaze positions of all samples of the same trial. This correction procedure is similar to the one used by Grosjean and colleagues [35]. Next, a 3D gaze point was calculated from the binocular 2D gaze positions. For this, we used the individual positions of the eyes, and the vector-based approach with the algorithm provided in the toolbox Gaze3DFix [48]. The 3D gaze points were used to detect fixations based on an ellipsoidal bounding volume as described in [48] with a duration threshold of 100 ms and a dispersion threshold of 2°. The position of the fixation containing the button press of the participants indicating that they looked at the center of the object was used as the vergence distance. Data were then transformed into an egocentric coordinate system with the unit cm. To calculate vergence velocity and vergence latency, the 3D gaze points were transformed to degree using the individual interpupillary distances. Vergence velocity was calculated using a two-point central difference algorithm and smoothed with a 15 Hz low pass filter. Vergence onset was defined as the first time after object onset when velocity exceeded 10°/s for at least 20 ms. Vergence latency was calculated as the time from object onset to vergence onset. Maximal vergence velocity was picked from the time interval between vergence onset and 650 ms after object onset. The procedures for obtaining vergence latency and maximal vergence velocity is similar to the approaches of other researchers [35, 50, 51].

Raw data from the motion tracker was corrected by the offset between the tip of the finger and the marker. The onset of the reaching movement was defined as the first time when the marker was lifted more than 2 cm above the button. Reaching duration was calculated as the time from onset of the reaching movement to the button press indicating the subjectively defined end position. To calculate the reaching distance, we used the sample containing the button press of the participant, 11 samples before and 12 samples after it and calculated the median. Since we measured reaching movements with 120 Hz, we thus averaged over 200 ms. Data were then transformed into an egocentric coordinate system with the unit cm.

In the process of data filtering, all trials, in which a participant did not follow the instructions (e.g., pressed the buttons in the wrong order), reported to have seen a double image, or in which there were technical problems, were excluded from the analyses (8%). For vergence movement analyses, trials containing a blink in the first 50 ms and trials with a vergence latency shorter than 50 ms were excluded as this distorts the baseline correction described above. Also, vergence distance had to be between 0 and 60 cm, vergence latency between 50 and 650 ms. Further, for vergence latency and maximal vergence velocity, trials containing a blink in the first 650 ms were excluded. For reaching movement analyses, we excluded trials in which the finger was still on the table when reaching distance was collected as apparent in the height of the marker on the fingertip. After data filtering, 86% of the trials remained for analyses in the relevant distances of 30 and 40 cm for vergence distance; 74% of the trials remained for vergence latency and maximal vergence velocity; 89% for reaching distance and reaching duration.

#### Data analysis

All analyses were done using linear mixed models (LMMs). The disparity and size manipulations were coded as factors named disparity-specified distance (30, 40) and familiar size-specified distance (30, 40), which could thus be congruent with no conflict between the two depth cues or incongruent with the two depth cues specifying different distances (conflict trials). Please note that this coding is equivalent to other studies [8, 10, 14]. For the dependent variable reaching duration, we compared congruent trials and conflict trials. We used log-transformed values for the dependent variables maximal vergence velocity and reaching duration to stabilize the error variance.

LMMs were used for the analyses since they allow to simultaneously account for multiple sources of (random) variability and thus generalize the experimental effects and interactions across both subjects and items [52]. Since we presented six different objects to 25 participants, we have by-item as well as by-subject and possibly even subject-by-item interaction variation in our data. In principle, each level of these three grouping factors can have an idiosyncratic mean response as well as idiosyncratic main effects and interactions. This can be modeled by including random intercepts and random slopes in the LMMs.

Choosing the appropriate random effects structure is crucial when using LMMs [53]. A maximal model does not only consider variance between subjects, between items, and between subject-item combinations in the mean of the dependent variable through random intercepts, but also variance between subjects, between items and between subject-item combinations for all within-unit fixed effects through random slopes as well as correlations between intercepts and slopes. Simulation studies have shown that maximal models minimize false positive results [53, 54]. As an example, the maximal model for the analysis of reaching distance is described as follows (using the formula notation of the R package lme4):

Reachingdistance~Reachingtype*Disparity-specifieddistance*Familiarsize-specifieddistance+(Reachingtype*Disparity-specifieddistance*Familiarsize-specifieddistance|Participant)+(Reachingtype*Disparity-specifieddistance*Familiarsize-specifieddistance|Object)+((Reachingtype+Disparity-specifieddistance+Familiarsize-specifieddistance)^2|Participant:Object)

We modeled the main effect of reaching type (sighted or blind reaches), the main effect of disparity-specified distance (30 or 40), the main effect of familiar size-specified distance (30 or 40) as well as all interactions between them as fixed effects. We then added random intercepts, random slopes, as well as correlations between them for participants, objects, and participant-object-combinations. For the three-way interaction there was no random slope for participant-object combinations, as each object was only presented once for each participant in each combination of conditions. For participants, random intercepts account for the fact that participants may differ in their general tendency to perceive distances—further away or closer; random slopes account for the fact that the manipulations (reaching type, disparity-specified distance, familiar size-specified distance) might affect some participants more than others (e.g., in previous studies participants differed in the weight they assigned to familiar size in their reaching estimates). For objects, random intercepts account for the fact that objects may differ in their general tendency to be perceived as closer or further away; random slopes account for the fact that the manipulations might have an effect for some objects but not for others. Random intercepts and random slopes for participant-object-combinations account for the fact that the mean reaching distance might vary between different participant-object-combinations and that the experimental effects might not be of the same size for all combinations of participants and objects. Since this is a study on familiar size, it is crucial that the participants recognize the object and activate a concept of its usual size but this is most likely not the case for all objects for all participants. For example, some participants might play tennis and know exactly the size of a tennis ball and this should influence their behavior; whereas those who have not seen or touched a tennis ball for years are not influenced by its size, but might be influenced by the size of other objects. Additionally, all correlations between random slopes and random intercepts within the same grouping factor can be modeled. This maximal model would require estimating a total of 23 variance components plus 77 correlations. To estimate this random effects structure, there are not enough data points in our study; therefore, the estimation algorithm does not converge when estimating a maximal model. Even if it would converge, the estimated LMMs would be still likely to be over-parameterized relative to the data and their estimated parameters thus not reliable. Therefore, we followed the recommendation by Bates and colleagues [55], started with the maximal model but reduced model complexity in an iterative process.

LMM analyses were estimated with orthogonal contrasts in R using the package afex [56], which is based on the package lme4 [57]. For model parameter estimation, restricted maximum likelihood (REML) estimation was used.

We always started with the maximal model. Since this does usually not converge (given the high number of model parameters that need to be estimated), in the next step, we specified a zero-correlation parameter model including all variance components, but no covariance parameters. We then did a principal component analysis (PCA) of the variance-covariance matrices of the random effects structure using the function rePCA() from the package RePsychLing [58]. When the number of dimensions needed for capturing 100% of the explained variance was lower than the number of specified variance components in the model, the model was further reduced by removing the variance component(s) with the smallest variability. As suggested by Bates and colleagues [55], this was repeated until the PCA no longer suggested overidentification. Also according to Bates and colleagues [55], in the next step, the model was further reduced by iteratively removing non-significant variance components. In each iteration, the further reduced model was compared with the one before with likelihood ratio tests (LRTs) comparing the goodness of fit. This was repeated until the reduction of the model led to a significant drop in the goodness of fit. As recommended by Matuschek and colleagues [59], we chose α_LRT_ = .2 as significance level for this model-selection process instead of using the standard α_LRT_ = .05 which would increase the Type I error rate. Eventually, we tested if the inclusion of covariance parameters significantly improved model fit. The final models can be found in S1 Table.

After model reduction, the final model was used to exclude outliers. As the presence of outliers may cause stress in the model and distort the estimated parameters, we removed data points with absolute standardized residuals greater than 3 (between 0.68 and 1.92% of the data).

After data reduction, significance values for the fixed effects of the final model were computed via the Kenward-Roger approximation for degrees of freedom [60]. Simulation studies have shown that the Kenward-Roger approximation produces nominal Type 1 error rates even for smaller samples [61]. As a effect sizes, we report marginal R squared (*R*_m_^2^) and conditional R squared (*R*_c_^2^) for the whole model as well as semi-partial marginal R squared ($R_{{\text{m}}_{\text{Sp}}}{}^{2}$) for each fixed effect in the model computed with the packages r2glmm [62] and MuMIn [63]. The packages are based on the method of Johnson [64], which is an extension to the method of Nakagawa and Schielzeth [65]. $R_{{\text{m}}_{\text{Sp}}}{}^{2}$ describes the variance explained by each fixed effect, R_m_^2^ describes the variance explained by all fixed effects in the model and R_c_^2^ describes the variance explained by all fixed effects and all random effects in the model together. For non-significant fixed effects effect sizes are not reported as all of them were vanishingly small ($R_{{\text{m}}_{\text{Sp}}}{}^{2}<.002$). Post hoc tests were done using the package emmeans [66] and *p*-values adjusted with the Holm-Bonferroni method. As a measure for covariance between continuous data, we report repeated measures correlations [67], calculated with the R package rmcorr [67]. All graphs were done using the R package ggplot2 [68].

### Results

#### Vergence distance and reaching distance in congruent trials for all distances

**Fig 2:**
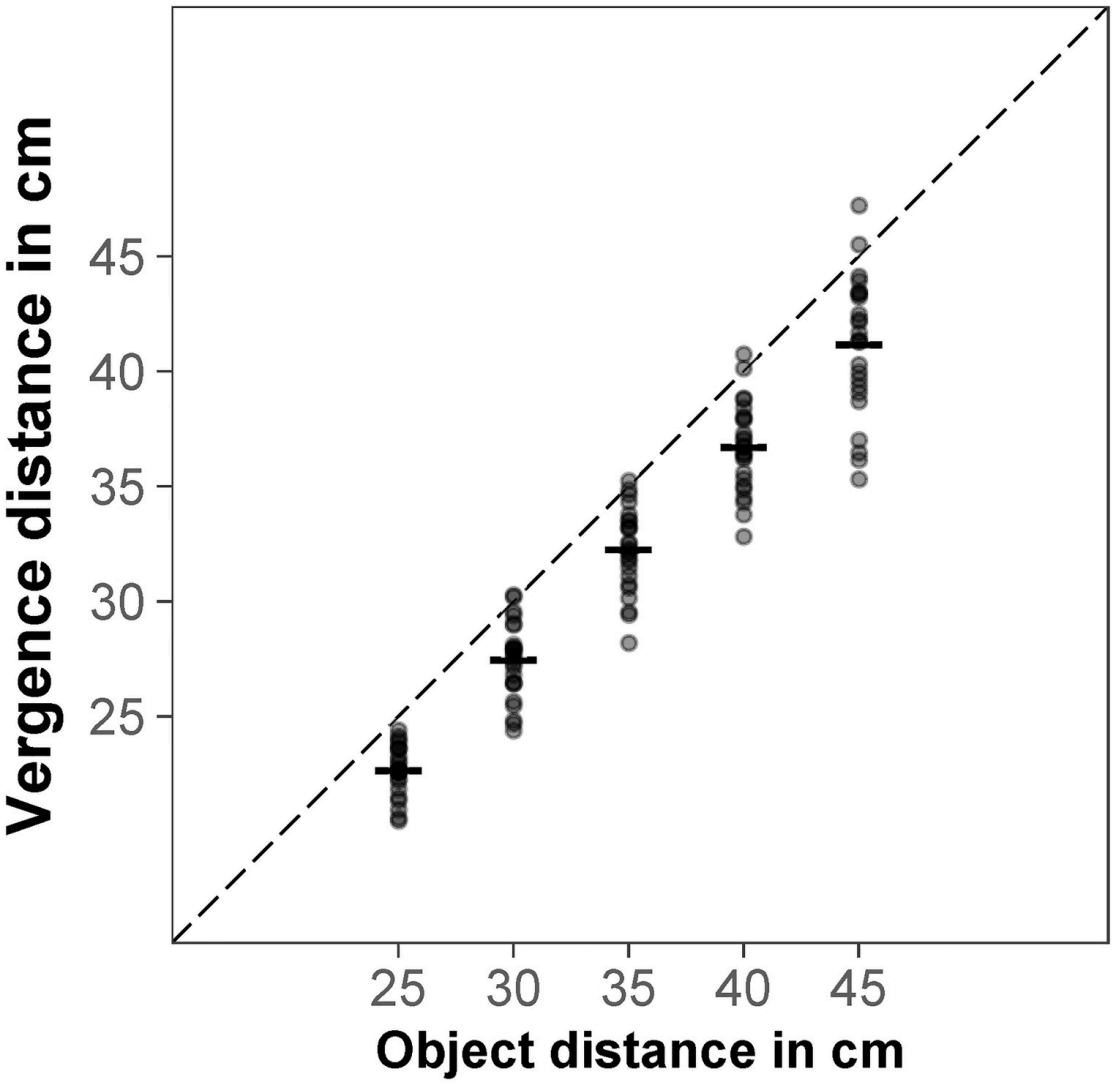
Vergence distance in congruent trials. Mean vergence distance for object distance; each data point represents the median for one participant (aggregated over six objects); the dashed black line represents the object distance as specified by binocular disparity and familiar size.

First of all, we looked at all congruent trials. We aggregated vergence distance and reaching distance over the six objects using the median. As expected, the repeated measures correlation indicated that there was a significant association between aggregated vergence distance and object distance, *r*_*rm*_ = .99, *p* < .001, the further away the object was presented, the further the participants looked (Fig 2).

**Fig 3:**
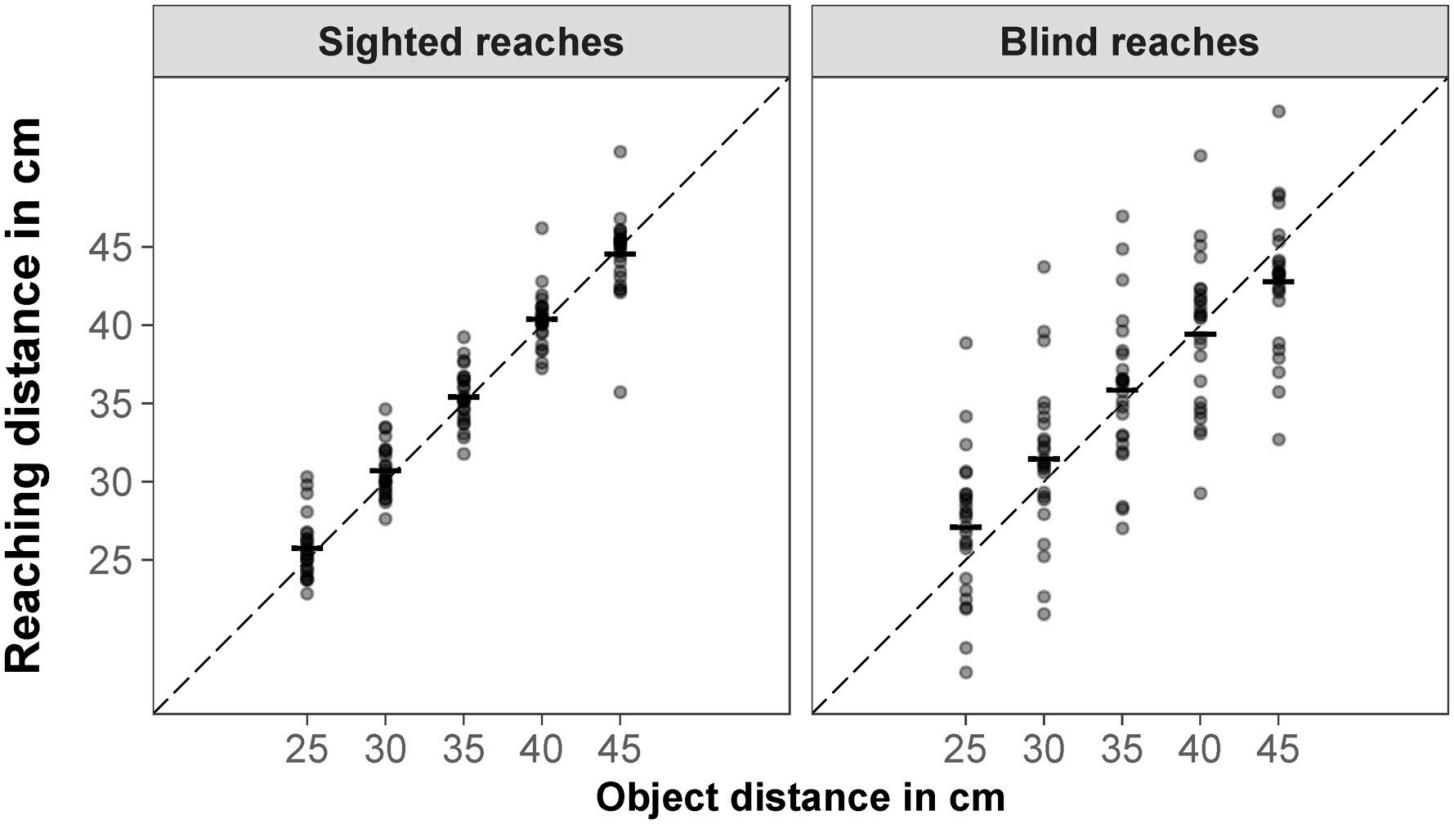
Reaching distance in congruent trials. Mean sighted and blind reaches for object distance; each data point represents the median for one participant (aggregated over six objects); the dashed black line represents the object distance as specified by binocular disparity and familiar size.

For reaching distance, there was a significant repeated measures correlation between aggregated reaching distance and object distance for sighted reaches, *r*_*rm*_ = .98, *p* < .001; the further away the object was presented, the further the participants reached. There was more interindividual variance in the aggregated reaching distances when participants reached without visual feedback (blind reaches). However, the repeated measures correlation was still high and significant for blind reaches, *r*_*rm*_ = .95, *p* < .001 (Fig 3).

#### Vergence movements in congruent and conflict trials at 30 and 40 cm

**Fig 4:**
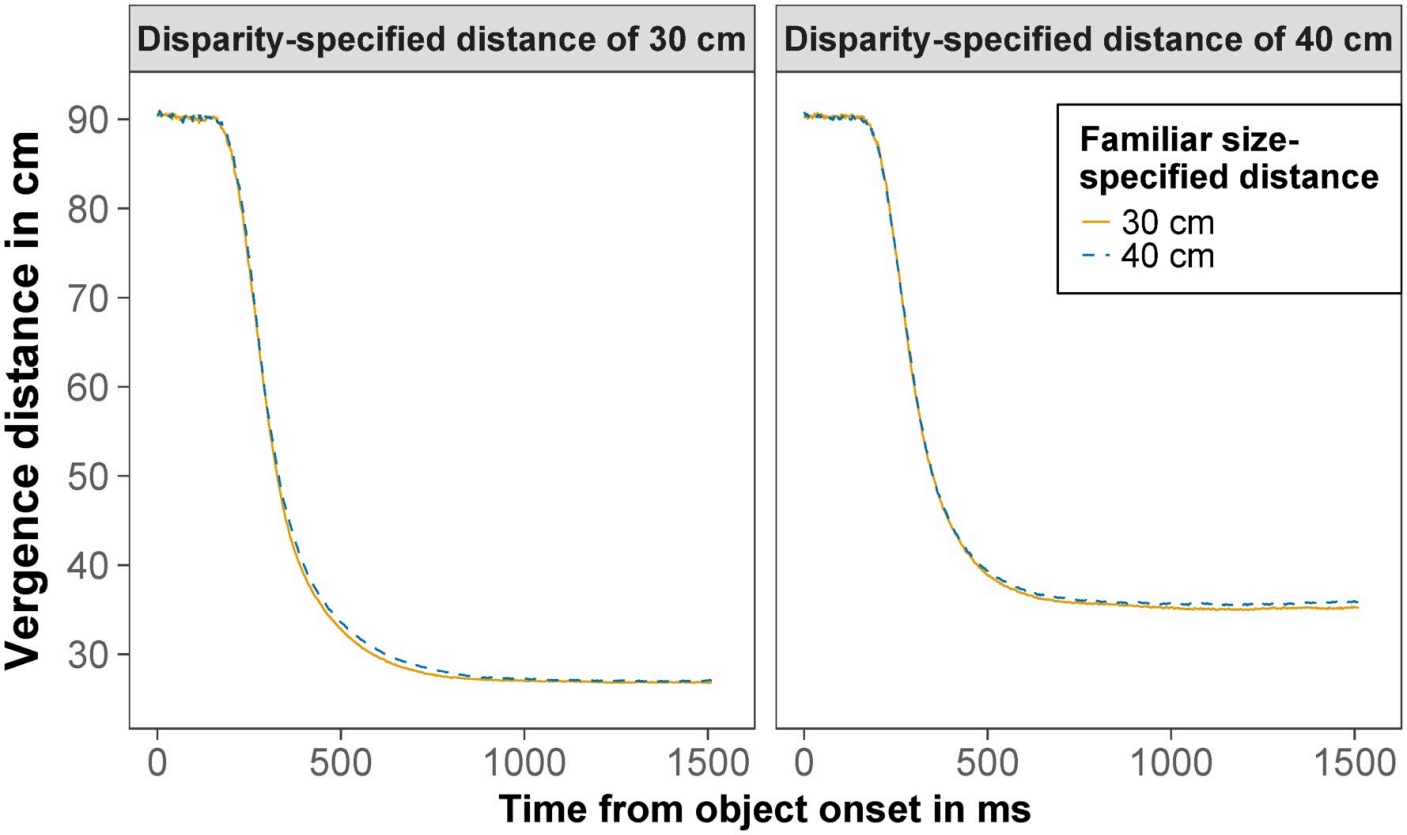
Vergence distance course. Mean vergence distance for a binocular disparity-specified distance of 30 cm (left) and 40 cm (right) and a familiar size-specified distance of 30 cm (solid orange) and 40 cm (dashed blue) as a function of time from object onset.

Next, we compared the vergence movements in congruent and conflict trials for the relevant distances of 30 and 40 cm. Fig 4 shows the mean vergence course for the four conditions: a disparity-specified distance of 30 cm with a familiar size-specified distance of 30 (congruent) or 40 cm (conflict) and a disparity-specified distance of 40 cm with a familiar size-specified distance of 30 (conflict) or 40 cm (congruent).

For vergence movements, the parameters vergence latency, maximal vergence velocity, and vergence distance were tested. Mean vergence latency, the time from object onset until the vergence movement started, was 216 ms. Using linear mixed models, we tested whether vergence latency was influenced by disparity-specified distance or familiar size-specified distance. Neither the main effect of disparity-specified distance, *F*(1,764.60) = 0.21, *p* = .651, nor the main effect of familiar size-specified distance, *F*(1,764.95) = 2.41, *p* = .121, nor the interaction between them, *F*(1,761.18) = 0.02, *p* = .885, was significant. All fixed effects together explained only 0.2% of the variance (*R*_m_^2^ = .002), the whole model explained 45% of the variance (*R*_c_^2^ = .454). Thus, vergence latency did not significantly depend on disparity-specified distance or familiar size-specified distance.

Next, we tested the effect of disparity-specified distance and familiar size-specified distance on maximal vergence velocity. The main effect of disparity-specified distance was significant, *F*(1,670.06) = 363.08, *p <* .001, $R_{{\text{m}}_{\text{Sp}}}{}^{2}=.189$, indicating that the maximal vergence velocity for a disparity-specified distance of 30 cm was higher (*M* = 47.67 °/s) than for a disparity-specified distance of 40 cm (*M* = 35.53 °/s). Neither the main effect of familiar size-specified distance, *F*(1,132.77) = 0.07, *p* = .799, nor the interaction, *F*(1,668.73) = 0.16, *p* = .685, was significant. All fixed effects in the model explained 19% of the variance (*R*_m_^2^ = .189), the whole model explained 55% of the variance (*R*_c_^2^ = .548).

**Fig 5:**
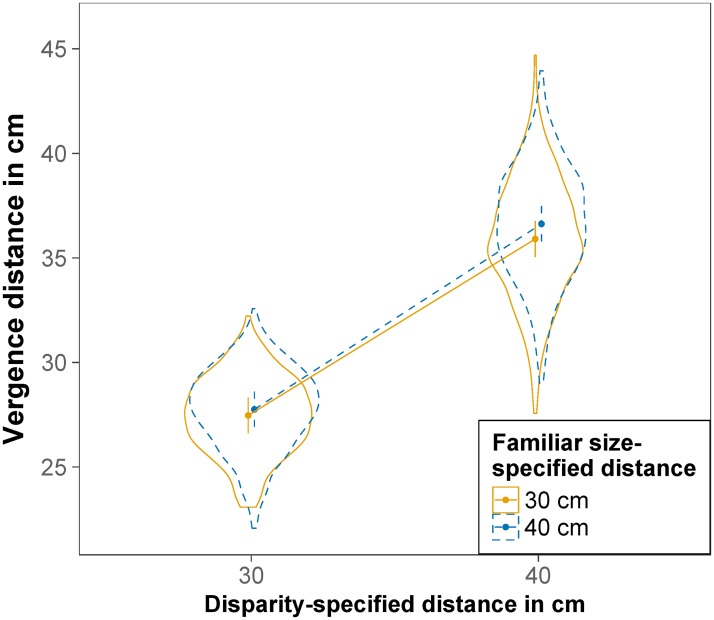
Vergence distance. Estimated marginal means of vergence distance for a binocular disparity-specified distance of 30 cm and 40 cm and a familiar size-specified distance of 30 cm (solid orange) and 40 cm (dashed blue). Error bars are model-based 95% confidence intervals indicating which values of the estimated means are likely and do not permit comparisons across repeated-measures factors [56]. Violin plots depict the distribution of the raw data.

The effect of disparity-specified distance and familiar size-specified distance on vergence distance is displayed in Fig 5. The main effect of disparity-specified distance was significant, *F*(1,23.96) = 2450.60, *p <* .001, $R_{{\text{m}}_{\text{Sp}}}{}^{2}=.762$, indicating that vergence distance was nearer for a disparity-specified distance of 30 cm (*M* = 27.66 cm) than for a disparity-specified distance of 40 cm (*M* = 36.24 cm). The main effect of familiar size-specified distance was also significant, *F*(1,968.17) = 24.83, *p* < .001, $R_{{\text{m}}_{\text{Sp}}}{}^{2}=.011$, indicating that vergence distance was nearer for a familiar size-specified distance of 30 cm (*M* = 31.66 cm) than for a familiar size-specified distance of 40 cm (*M* = 32.28 cm). The interaction was also significant *F*(1,968.25) = 4.53, *p* = .034, $R_{{\text{m}}_{\text{Sp}}}{}^{2}=.002$, indicating a smaller effect of familiar size-specified distance at a disparity-specified distance of 30 cm compared to 40 cm. Contrasts revealed that the effect of familiar size-specified distance on vergence distance was significant both for a disparity-specified distance of 30 cm, *t*(967.85) = -2.02, *p* = .044, and a disparity-specified distance of 40 cm, *t*(968.57) = -5.03, *p* < .001. All fixed effects of the model explained 76% of the variance (*R*_m_^2^ = .763), the whole model explained 89% of the variance (*R*_c_^2^ = .895).

#### Reaching movements in congruent and conflict trials at 30 and 40 cm

**Fig 6:**
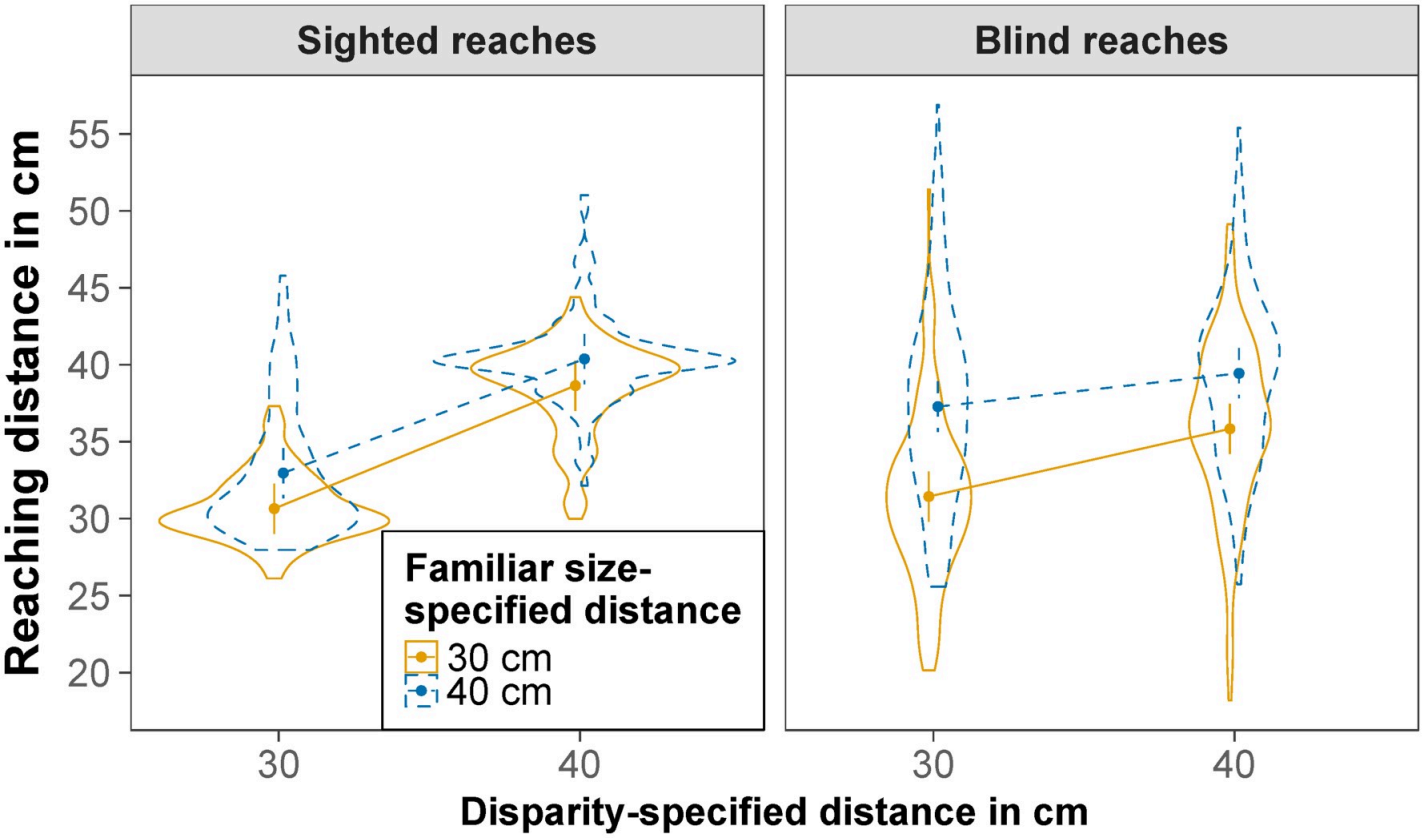
Reaching distance. Estimated marginal means of reaching distance for sighted reaches (left) and blind reaches (right), for a binocular disparity-specified distance of 30 cm and 40 cm and a familiar size-specified distance of 30 cm (solid orange) and 40 cm (dashed blue). Error bars are model-based 95% confidence intervals indicating which values of the estimated means are likely and do not permit comparisons across repeated-measures factors [56]. Violin plots depict the distribution of the raw data.

**Fig 7:**
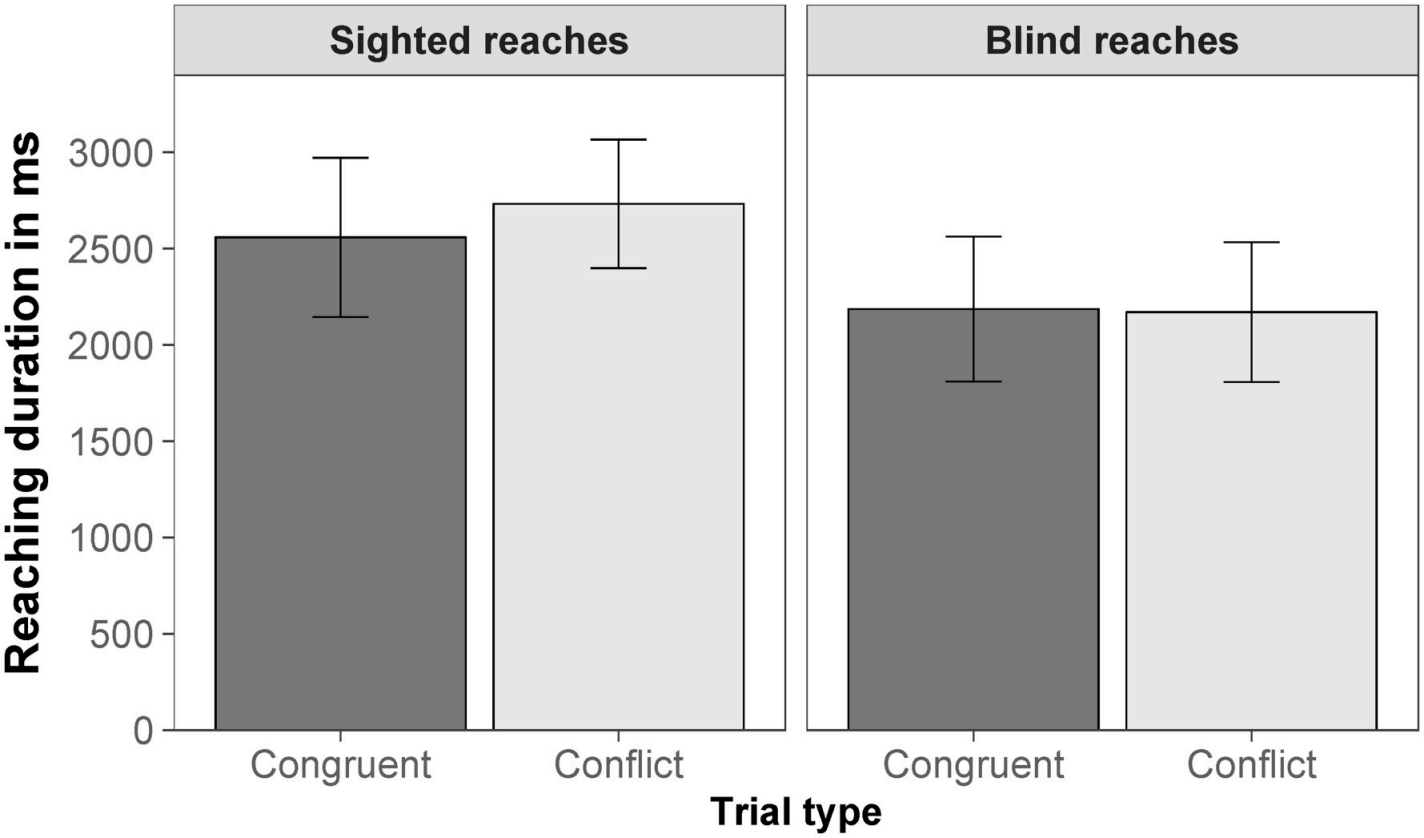
Reaching duration. Mean reaching duration for sighted reaches (left) and blind reaches (right) for congruent trials (disparity-specified distance corresponded to familiar size-specified distance; dark gray) and conflict trials (disparity-specified distance was opposed to familiar size-specified distance; light gray) of the by-subject aggregated data. Error bars are within-subjects 95% confidence intervals according to Morey [69].

Next, we compared reaching distance and reaching duration in congruent and conflict trials for the relevant distances of 30 and 40 cm. The effect of disparity-specified distance and familiar size-specified distance on reaching distance is displayed in Fig 6. The main effect of reaching type was not significant, *F*(1,24.00) = 0.18, *p* = .678. The main effect of disparity-specified distance was significant, *F*(1,23.99) = 122.40, *p* < .001, $R_{{\text{m}}_{\text{Sp}}}{}^{2}=.268$, indicating that reaching distance was nearer for a disparity-specified distance of 30 cm (*M* = 33.31 cm) than for a disparity-specified distance of 40 cm (*M* = 38.65 cm). The main effect of familiar size-specified distance was also significant, *F*(1,24.00) = 75.63, *p* < .001, $R_{{\text{m}}_{\text{Sp}}}{}^{2}=.121$, indicating that reaching distance was nearer for a familiar size-specified distance of 30 cm (*M* = 34.24 cm) than for a familiar size-specified distance of 40 cm (*M* = 37.68 cm). The interaction between reaching type and disparity-specified distance was also significant *F*(1,23.89) = 106.78, *p* < .001, $R_{{\text{m}}_{\text{Sp}}}{}^{2}=0.56$, indicating a larger effect of disparity-specified distance for sighted as compared to blind reaches. The interaction between reaching type and familiar size-specified distance was also significant *F*(1,23.83) = 53.03, *p* < .001, $R_{{\text{m}}_{\text{Sp}}}{}^{2}=0.22$, indicating a smaller effect of familiar size-specified distance for sighted as compared to blind reaches. The interaction between disparity-specified distance and familiar size-specified distance was also significant *F*(1,145.01) = 24.02, *p* < .001, $R_{{\text{m}}_{\text{Sp}}}{}^{2}=.006$, indicating a larger effect of familiar size-specified distance for a disparity-specified distance of 30 cm as compared to 40 cm. The significant three-way interaction, *F*(1,260.25) = 8.93, *p* = .003, $R_{{\text{m}}_{\text{Sp}}}{}^{2}=.002$, indicated that this difference is mainly true for blind reaches. Contrasts revealed that the effect of familiar size-specified distance on reaching distance was significant for all four combinations of conditions (all *t*s < -3.69, all *p*s < .001) (Fig 7). All fixed effects of the model explained 38% of the variance (*R*_m_^2^ = .376), the whole model explained 85% of the variance (*R*_c_^2^ = .851). There was a negative Pearson correlation between the participants’ random slopes (or, more precisely, best linear unbiased predictions) for disparity-specified distance and participants’ random slopes for familiar size-specified distance, *r* = -.85, *p* < .001; the more weight a participant gave to binocular disparity, the less weight he or she gave to familiar size.

Reaching duration, the time from the start of the reaching movement until the button press indicating the end of the movement, was on average 2738 ms for sighted reaches and 2232 ms for blind reaches (Fig 7). We tested whether reaching duration was different for congruent trials (disparity-specified distance corresponded to familiar size-specified distance) and conflict trials (disparity-specified distance was opposed to familiar size-specified distance). The main effect of reaching type was significant, *F*(1,25.48) = 4.33, *p* = .048, $R_{{\text{m}}_{\text{Sp}}}{}^{2}=.022$. The main effect of trial type was significant, *F*(1,892.04) = 15.22, *p* < .001, $R_{{\text{m}}_{\text{Sp}}}{}^{2}=.004$, indicating that reaching duration was longer for conflict trials (*M* = 2576 ms) compared to congruent trials (*M* = 2407 ms). The interaction between reaching type and trial type was also significant, *F*(1,23.73) = 9.75, *p* = .005, $R_{{\text{m}}_{\text{Sp}}}{}^{2}=.003$, indicating that the difference between congruent and conflict trials was larger and significant for sighted reaches, *t*(79.59) = 5.01, *p* < .001, but smaller and not significant for blind reaches, *t*(83.86) = 0.39, *p* = .698. All fixed effects of the model explained 3% of the variance (*R*_m_^2^ = .028), the whole model explained 76% of the variance (*R*_c_^2^ = .764).

### Discussion

The research question was whether familiar size is considered for controlling reaching and vergence movements. To investigate this, we systematically varied the size of stereoscopically presented familiar everyday objects while measuring vergence eye movements and reaching movements.

Concerning reaching movements, we found that participants reached in the expected distance when binocular disparity and familiar size specified the same distance (congruent trials). When the two depth cues specified different distances (conflict trials), both binocular disparity and familiar size significantly influenced reaching distance. When visual feedback was available (sighted reaches), binocular disparity was given more weight while without visual feedback (blind reaches) reaching distances were more affected by familiar size. Thus, the results suggest that both depth cues were combined with the weight depending on the reaching type (with or without visual feedback) and the participant’s strategy. These results are in line with the findings of an earlier experiment [19], in which we found that binocular disparity and familiar size were both used for reaching movements towards a virtual tennis ball.

Concerning vergence movements, the mean of the vergence distances in congruent trials was smaller than expected for all distances. On average, computed vergence distance was about 3 cm shorter than object distance as specified by binocular disparity and familiar size. On the one hand, this is not surprising as other researchers have also found non-perfect vergence distances and considerable individual differences [35, 70–72]. On the other hand, however, we had measured more precise vergence distances in earlier experiments using the same toolbox [48]. In our experiment, there are several possible sources for measurement errors: First, the shutter glasses in combination with the infrared light source of the motion tracking system might have reduced eye tracking quality. Second, the distance from the monitor to the participant was measured to the pupil of the participant instead to the retina. Third, we used a binocular calibration procedure; there is a debate as to whether using a monocular calibration should be preferred when studying binocular eye movements [73–75]. Fourth, just after finishing our experiment, Hooge and colleagues [76] published their work showing that changes in pupil size evoked by changes in the luminance of the stimulus can lead to systematic changes in measured vergence distance. The reason is that a change in pupil size may be accompanied by a change in pupil shape resulting in a shift of the location of the center of gravity of the pupil, which results in the eye-tracker reporting a change in gaze direction [76]. These authors used the same eye-tracking technology as we did in our experiment. And indeed, we also found significant correlations between pupil size and vergence distance at a disparity-specified distance of 30 cm, *r*_*rm*_ = .44, *p* < .001, and 40 cm, *r*_*rm*_ = .31, *p* < .001. Thus, we found evidence for the pupil size artefact in our data in line with Hooge and colleagues [76] and the deviating vergence distance in congruent trials might therefore be due to changes in luminance as the calibration screen was darker compared to the stimulus.

When familiar size-specified distance was in conflict with disparity-specified distance, vergence distance followed largely the distance as specified by binocular disparity. This is in line with models on the control of vergence movements [23, 24] and experimental studies showing that vergence follows binocular disparity [30–32]. However, the effect of familiar size-specified distance on vergence distance was also significant. This means that in the conflict trials vergence distance was slightly deviated towards the conflicting distance as specified by familiar size. This effect was small: roughly 4 mm for a disparity-specified distance of 30 cm and about 8 mm for a disparity-specified distance of 40 cm. This result could be interpreted in the way that familiar size is taken into account for the control of vergence movements. This would be in line with other research showing that vergence movements are not solely based on binocular disparity but that other depth cues can be taken into account when programming vergence movements [28, 33–38]. However, the effect might not be due to the use of familiar size as a depth cue but might be a measurement error due to the pupil size artefact. Since we presented the objects on a dark background, a larger stimulus did not only suggest a smaller distance towards it, but had also more luminance. Thus, a larger stimulus could result in pupil size changes and subsequently to measurement errors in the vergence distance according to Hooge and colleagues [76]. To rule out the possibility that the effect of familiar size on vergence distance is not due to familiar size itself but primarily due to changing luminance we decided to replicate the experiment. In experiment 2, we used a light stimuli background in a bright environment—evoking a small pupil and reducing pupil size changes—as recommended by Hooge and colleagues [76].

## Experiment 2

### Methods

Experiment 2 was basically a replication of experiment 1. As in experiment 1, we systematically varied the size of familiar everyday objects while measuring vergence eye movements and reaching movements.

#### Participants

We conducted a power analysis based on simulations from an LMM (using the R package simr [77] in which the subject-mean centered pupil size was included as a covariate to partial out the potential within-subject association between pupil size and vergence distance [78]. The results showed that in order to replicate the effect of familiar size-specified distance on vergence distance with a power of at least .8 a sample size of at least 35 participants was necessary. Therefore, data from 36 participants were obtained. All participants were right-handed as indicated by the Edinburgh Handedness Inventory [39] and passed the same visual tests as described for experiment 1. Participants were aged between 19 and 47 years (*M* = 24.25, *SD* = 5.79); 27 of them were women, 9 were men. Participants were naive to the purpose of the study. Written informed consent was obtained prior to the study. Participants were compensated for their participation with either € 12 or course credit. The experimental procedure was approved by the local ethics committee (vote EK 315082014 of the ethics committee of the Technische Universität Dresden, Germany).

#### Setup, stimuli, procedure, data preparation, and data analysis

The experimental setup was exactly the same as in experiment 1 except for the lighting conditions. While experiment 1 was conducted in a dimly lit room, all lights were turned on in experiment 2 resulting in a brightly lit room. The stimuli were the same as in experiment 1 with the exception that the six objects were presented on a bright background. The procedure of the experiment and the instructions for the participants were exactly the same as in experiment 1. Data preparation was also exactly the same as in experiment 1. After data filtering, 88% of the trials remained for analyses in the relevant distances of 30 and 40 cm for vergence distance; 75% of the trials remained for vergence latency and maximal vergence velocity; 89% for reaching distance and reaching duration. Data were analyzed using LMMs with the procedure described above. For comparability to experiment 1, we used log-transformed values for the dependent variables maximal vergence velocity and reaching duration. The final models can be found in S1 Table.

### Results

Results for experiment 2 are reported following the same structure as for experiment 1.

#### Vergence distance and reaching distance in congruent trials for all distances

**Fig 8:**
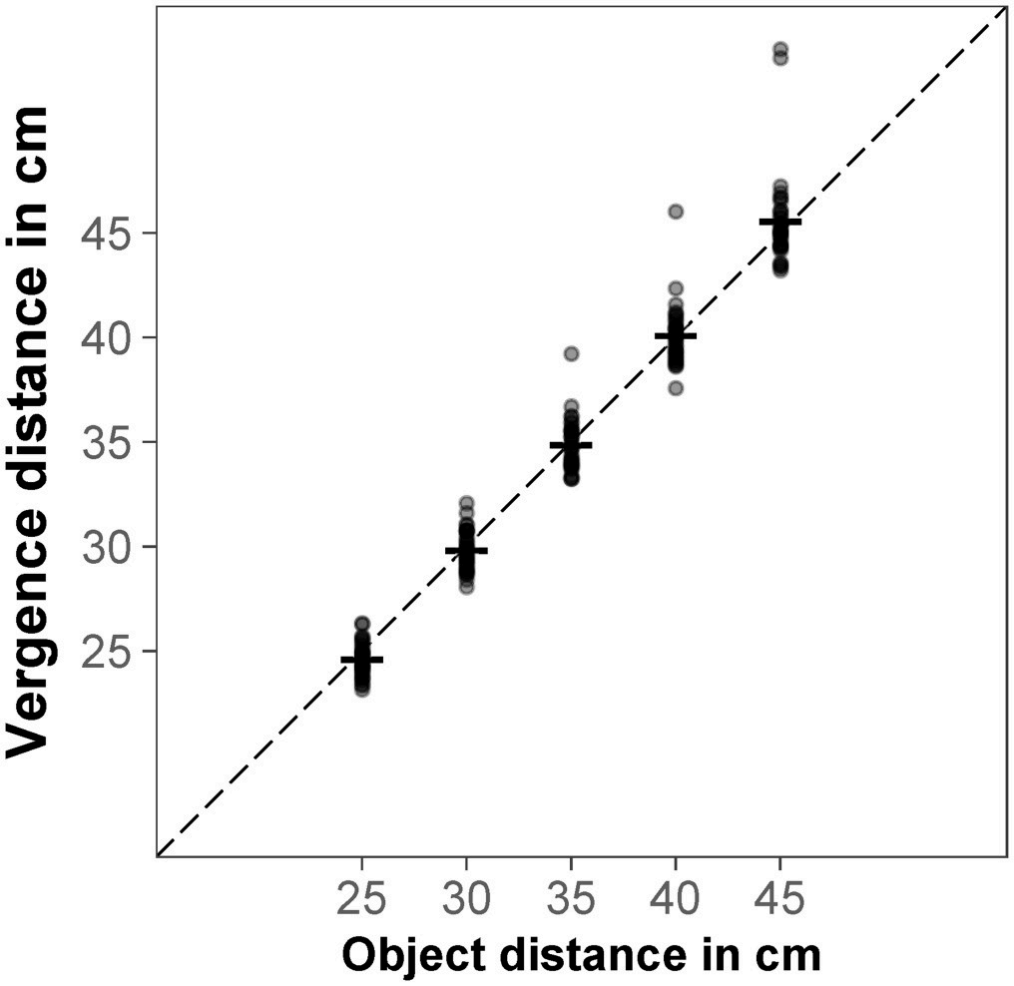
Vergence distance in congruent trials. Mean vergence distance for object distance; each data point represents the median for one participant (aggregated over six objects); the dashed black line represents the object distance as specified by binocular disparity and familiar size.

For congruent trials, repeated measures correlation indicated that there was a significant association between aggregated vergence distance and object distance, *r*_*rm*_ = .99, *p* < .001, the further away the object was presented, the further the participants looked (Fig 8).

**Fig 9:**
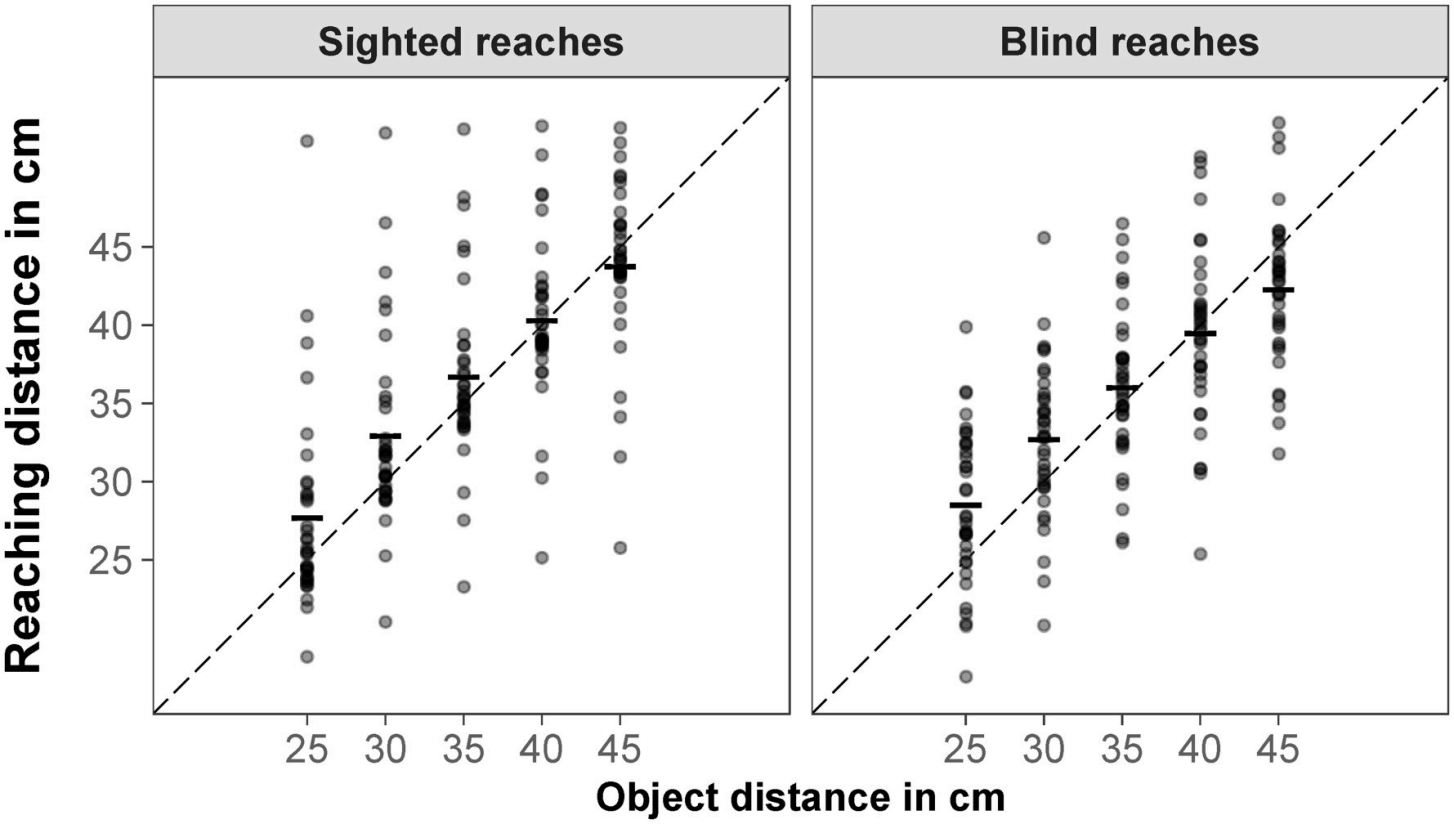
Reaching distance in congruent trials. Mean sighted and blind reaches for object distance; each data point represents the median for one participant (aggregated over six objects); the dashed black line represents the object distance as specified by binocular disparity and familiar size.

For reaching distance, there was a significant repeated measures correlation between aggregated reaching distance and object distance for sighted reaches, *r*_*rm*_ = .94, *p* < .001; the further away the object was presented, the further the participants reached. When participants reached without visual feedback (blind reaches), the repeated measures correlation was also high and significant, *r*_*rm*_ = .93, *p* < .001 (Fig 9).

#### Vergence movements in congruent and conflict trials at 30 and 40 cm

Next, we compared the vergence movements in congruent and conflict trials for the relevant distances of 30 and 40 cm. Mean vergence latency, the time from object onset until the vergence movement started, was 227 ms. We tested the effect of disparity-specified distance and familiar size-specified distance on vergence latency. Vergence latency was not significantly affected by disparity-specified distance, *F*(1,193.36) = 0.04, *p* = .836. There was a small, but significant effect of familiar size-specified distance, *F*(1,33.11) = 6.55, *p* = .015, $R_{{\text{m}}_{\text{Sp}}}{}^{2}=.004$, on vergence latency indicating that the vergence movement started 2 ms faster for a familiar-size specified distance of 30 cm (*M* = 226 ms) compared to 40 cm (*M* = 228 ms). The interaction between disparity-specified distance and familiar size-specified distance was not significant, *F*(1,1089.96) < 0.01, *p* = .921. All fixed effects together explained only 0.4% of the variance (*R*_m_^2^ = .004), the whole model explained 60% of the variance (*R*_c_^2^ = .601).

Next, we tested the effect of disparity-specified distance and familiar size-specified distance on maximal vergence velocity. The main effect of disparity-specified distance was significant, *F*(1,32.56) = 397.81, *p* < .001, $R_{{\text{m}}_{\text{Sp}}}{}^{2}=.157$, indicating that the maximal vergence velocity for a disparity-specified distance of 30 cm was higher (*M* = 46.42 °/s) than for a disparity-specified distance of 40 cm (*M* = 35.76 °/s). The main effect of familiar size-specified distance was also significant, *F*(1,1107.44) = 8.37, *p* = .004, $R_{{\text{m}}_{\text{Sp}}}{}^{2}=.003$, indicating that the maximal vergence velocity for a familiar size-specified distance of 30 cm was higher (*M* = 41.47 °/s) than for a familiar size-specified distance of 40 cm (*M* = 40.55 °/s). The interaction was not significant, *F*(1,1115.31) = 0.14, *p* = .706. All fixed effects in the model explained 16% of the variance (*R*_m_^2^ = .159), the whole model explained 62% of the variance (*R*_c_^2^ = .624).

**Fig 10:**
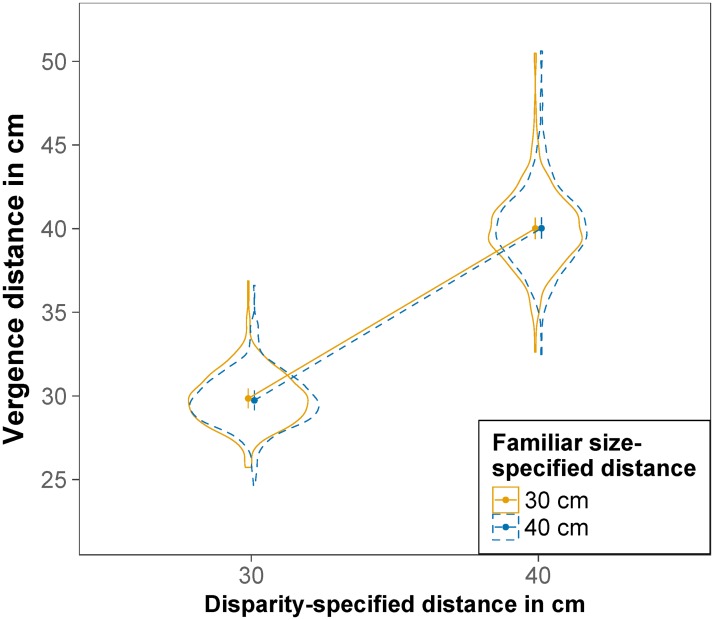
Vergence distance. Estimated marginal means of vergence distance for a binocular disparity-specified distance of 30 cm and 40 cm and a familiar size-specified distance of 30 cm (solid orange) and 40 cm (dashed blue). Error bars are model-based 95% confidence intervals indicating which values of the estimated means are likely and do not permit comparisons across repeated-measures factors [56]. Violin plots depict the distribution of the raw data.

The effect of disparity-specified distance and familiar size-specified distance on vergence distance is displayed in Fig 10. The main effect of disparity-specified distance was significant, *F*(1,33.95) = 6200.44, *p* < .001, $R_{{\text{m}}_{\text{Sp}}}{}^{2}=.87$, indicating that vergence distance was nearer for a disparity-specified distance of 30 cm (*M* = 29.79 cm) than for a disparity-specified distance of 40 cm (*M* = 40.05 cm). The main effect of familiar size-specified distance was not significant, *F*(1,1144.63) = 0.47, *p* = .493, nor was the interaction, *F*(1,1143.79) = 0.71, *p* = .399. All fixed effects of the model explained 87% of the variance (*R*_m_^2^ = .870), the whole model explained 93% of the variance (*R*_c_^2^ = .927).

#### Reaching movements in congruent and conflict trials at 30 and 40 cm

**Fig 11:**
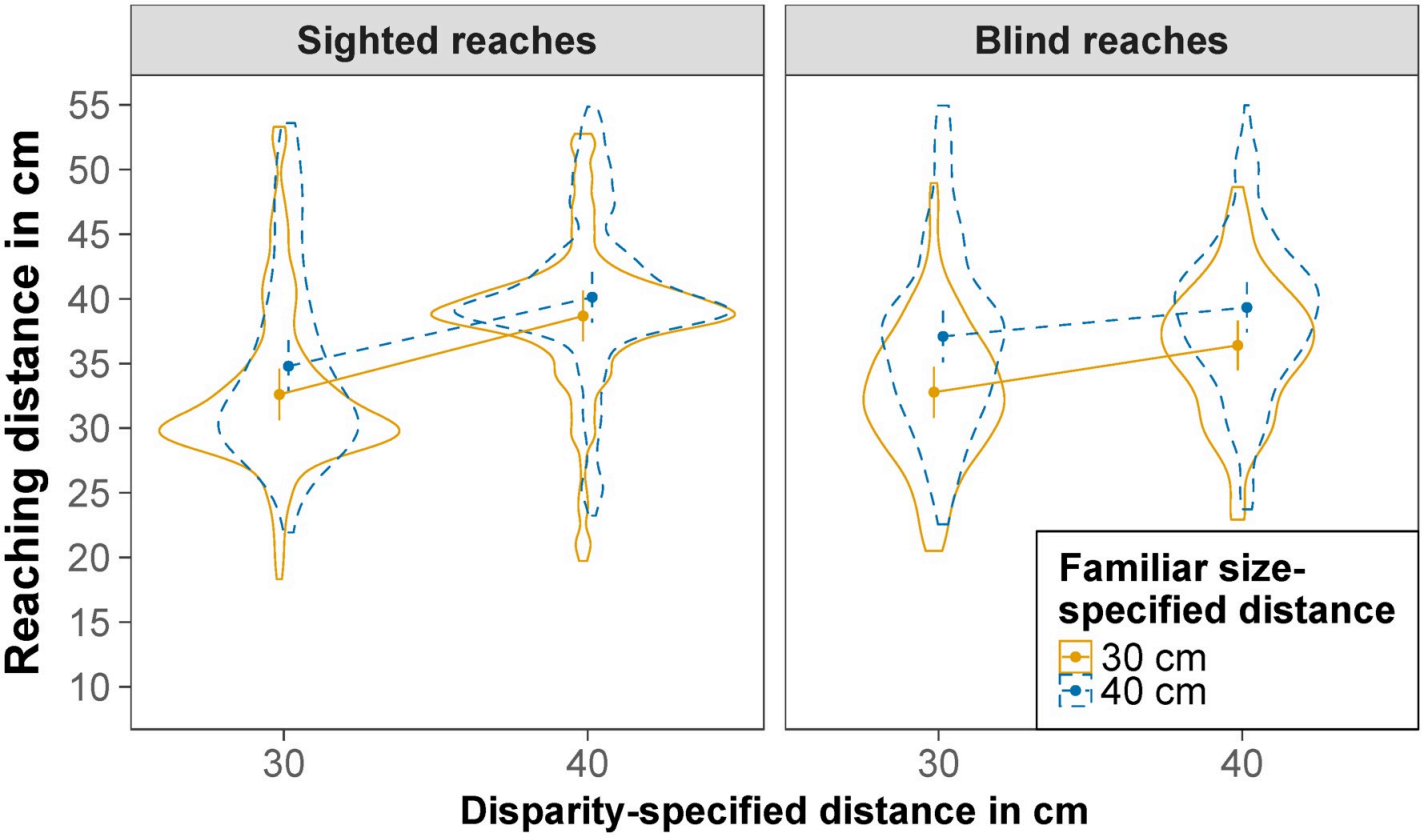
Reaching distance. Estimated marginal means of reaching distance for sighted reaches (left) and blind reaches (right), for a binocular disparity-specified distance of 30 cm and 40 cm and a familiar size-specified distance of 30 cm (solid orange) and 40 cm (dashed blue). Error bars are model-based 95% confidence intervals indicating which values of the estimated means are likely and do not permit comparisons across repeated-measures factors [56]. Violin plots depict the distribution of the raw data.

**Fig 12:**
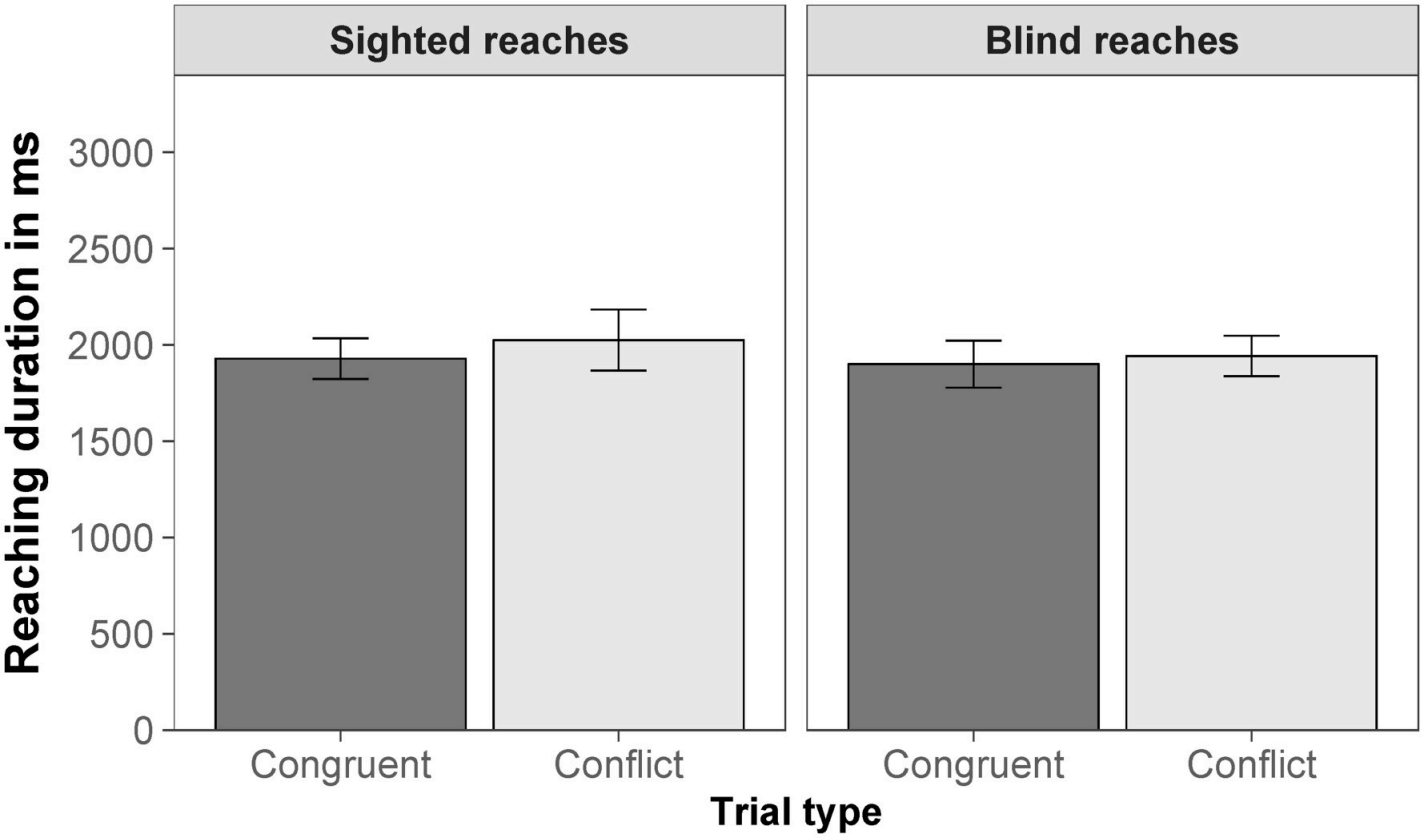
Reaching duration. Mean reaching duration for sighted reaches (left) and blind reaches (right) for congruent trials (disparity-specified distance corresponded to familiar size-specified distance; dark gray) and conflict trials (disparity-specified distance was opposed to familiar size-specified distance; light gray) of the by-subject aggregated data. Error bars are within-subjects 95% confidence intervals according to Morey [69].

Next, we compared reaching distance and reaching duration in congruent and conflict trials for the relevant distances of 30 and 40 cm. The effect of disparity-specified distance and familiar size-specified distance on reaching distance is displayed in Fig 11. The main effect of reaching type was not significant, *F*(1,34.31) = 0.04, *p* = .849. The main effect of disparity-specified distance was significant, *F*(1,36.18) = 81.35, *p* < .001, $R_{{\text{m}}_{\text{Sp}}}{}^{2}=.112$, indicating that reaching distance was nearer for a disparity-specified distance of 30 cm (*M* = 34.27 cm) than for a disparity-specified distance of 40 cm (*M* = 38.50 cm). The main effect of familiar size-specified distance was also significant, *F*(1,34.94) = 62.63, *p* < .001, $R_{{\text{m}}_{\text{Sp}}}{}^{2}=.048$, indicating that reaching distance was nearer for a familiar size-specified distance of 30 cm (*M* = 34.97 cm) than for a familiar size-specified distance of 40 cm (*M* = 37.84 cm). The interaction between reaching type and disparity-specified distance was also significant *F*(1,34.12) = 52.62, *p* < .001, $R_{{\text{m}}_{\text{Sp}}}{}^{2}=.013$, indicating a larger effect of disparity-specified distance for sighted as compared to blind reaches. The interaction between reaching type and familiar size-specified distance was also significant *F*(1,34.20) = 24.81, *p* < .001, $R_{{\text{m}}_{\text{Sp}}}{}^{2}=.005$, indicating a smaller effect of familiar size-specified distance for sighted as compared to blind reaches. The interaction between disparity-specified distance and familiar size-specified distance was also significant *F*(1,207.43) = 23.24, *p* < .001, $R_{{\text{m}}_{\text{Sp}}}{}^{2}=.002$, indicating a larger effect of familiar size-specified distance for a disparity-specified distance of 30 cm as compared to 40 cm. The three-way interaction, *F*(1,34.48) = 1.46, *p* = .235, did not reach significance. Contrasts revealed that the effect of familiar size-specified distance on reaching distance was significant for all four combinations of conditions (all *t*s < -3.42, all *p*s < .002) (Fig 12). All fixed effects of the model explained 17% of the variance (*R*_m_^2^ = .166), the whole model explained 91% of the variance (*R*_c_^2^ = .908). There was a negative Pearson correlation between the participants’ random slopes (or, more precisely, best linear unbiased predictions) for disparity-specified distance and participants’ random slopes for familiar size-specified distance, *r* = -.36, *p* = .030; the more weight a participant gave to binocular disparity, the less weight he or she tended to give to familiar size.

Reaching duration, the time from the start of the reaching movement until the button press indicating the end of the movement, was on average 2049 ms for sighted reaches and 1971 ms for blind reaches (Fig 12). We tested whether reaching duration was different for congruent trials (disparity-specified distance corresponded to familiar size-specified distance) and conflict trials (disparity-specified distance was opposed to familiar size-specified distance). The main effect of reaching type was not significant, *F*(1,34.25) = 0.05, *p* = .830. The main effect of trial type was significant, *F*(1,1304.13) = 8.26, *p* = .004, $R_{{\text{m}}_{\text{Sp}}}{}^{2}=.002$, indicating that reaching duration was longer for conflict trials (*M* = 2046 ms) compared to congruent trials (*M* = 1975 ms). The interaction between reaching type and trial type was not significant, *F*(1,209.72) = 0.44, *p* = .506. Contrasts revealed that the difference between congruent and conflict trials was significant for sighted reaches, *t*(629.73) = 2.46, *p* = .029, but not significant for blind reaches, *t*(646.52) = 1.48, *p* = .139. All fixed effects of the model explained less than 1% of the variance (*R*_m_^2^ = .002), the whole model explained 62% of the variance (*R*_c_^2^ = .616).

### Discussion

The aim of experiment 2 was to replicate the results of experiment 1 concerning reaching movements and to test the possibility that the significant effect of familiar size on vergence distance found in experiment 1 was not due to familiar size itself but a measurement error caused by the pupil size artefact [76]. Therefore, experiment 2 was a replication of experiment 1 differing only in luminance conditions. In experiment 2, we used a light stimulus background in a bright environment to evoke a small pupil and reduce pupil size changes. Again, we systematically varied the size of familiar everyday objects while measuring vergence eye movements and reaching movements.

Concerning reaching movements, we found that the mean reaching distances corresponded to the expected distance when binocular disparity and familiar size specified the same distance (congruent trials). Both binocular disparity and familiar size significantly influenced reaching distance when the two depth cues specified different distances (conflict trials). The results replicate the findings of experiment 1 and of a previously published experiment [19].

Concerning vergence movements, the mean of the vergence distances in congruent trials corresponded quite precisely to the expected distances. When familiar size-specified distance was in conflict with disparity-specified distance, vergence distance was significantly affected by binocular disparity. The effect of familiar size-specified distance was far from reaching significance and only explained less than 1% of the variance. Thus, the change in luminance conditions compared to experiment 1 did change the results concerning vergence movements. This suggests that the effect of familiar size-specified distance on vergence distance found in experiment 1 indeed has to be attributed to the pupil size artefact.

## General discussion

The size of familiar objects can be used as a cue to their distance. The influence of familiar size on relative depth perception and grasping movements has been shown. Here, two experiments were conducted to examine whether familiar size is taken into account for the control of reaching and vergence movements. Six different objects with familiar size were stereoscopically presented. The distance to the objects as specified by binocular disparity and the distance as specified by familiar size were manipulated. Participants made vergence movements and reaching movements with eyes open (sighted reaches) or with eyes closed (blind reaches) towards the objects. We begin with a discussion of the influence of familiar size on reaching movements before elaborating on the influence on vergence movements which is followed by a discussion of the limitations of the current experiment as well as open research questions.

### Familiar size influences reaching movements

In both experiments, reaching distances for sighted reaches were highly correlated with object distance as specified by binocular disparity and familiar size (congruent trials) and distributed around the expected values. This supports the basic assumption that most participants in the given setting are indeed able to reach correctly to stereoscopically presented objects. When no visual feedback was available during the reaching movement (blind reaches), reaching distances were more dispersed between subjects, but still highly correlated with object distance. This is in line with previous work demonstrating larger variation and more errors when visual feedback is not available [79–82]. For sighted and for blind reaches, there was a slight tendency to overestimate shorter distances and underestimate farther distances. This tendency towards the mean was stronger for blind reaches. The same bias, often more pronounced, has been found in other studies [7, 83–85].

When familiar size-specified distance was in conflict with disparity-specified distance, results from both experiments suggest that information from both depth cues was combined for reaching movements. Since we did not find evidence for bimodal distributions with means close to 30 and 40, respectively, of reaching distances, participants did likely not decide which depth cue to follow but made a compound distance estimate. Participants varied in the weight they gave to each cue for this compound estimate as indicated by the variance of the random slope of familiar size-specified distance for participants. While some participants oriented themselves more on binocular disparity, others gave more weight to familiar size. These results replicate the findings of an earlier experiment [19], in which we found that binocular disparity and familiar size were both used for reaching movements towards a virtual tennis ball with participants differing in the weight they assigned to each depth cue.

Also in line with our earlier experiment [19], we found that the effect of familiar size was larger for blind reaches. It is likely that familiar size was given considerable weight for planning the reaching movements for blind as well as for sighted reaches. For sighted reaches where visual feedback was available during the movement, however, information from binocular disparity was used to correct the reaching movement towards the distance as specified by disparity. This assumption is supported by the result that participants took more time to complete their reaches for sighted as compared to blind reaches. Further, for sighted reaches, reaching duration was longer for conflict trials as compared to congruent trials. This is in line with the two-component model [86], which assumes a ballistic, preprogrammed phase to bring the finger near the target and a homing phase during which feedback is used for final adjustments of the movement. According to this model, slower movements are more accurate because they allow more time for corrections during the homing phase. The results are also in line with the more recent multiple-process model of limb control [87], which builds upon the two-component model. It postulates that visual feedback is used from very early in the reaching movement until the end of the movement to correct the initial movement impulse. However, it takes time to pick up and utilize visual feedback and thus, movements are prolonged when visual feedback is used.

To sum up, familiar size influences reaching movements towards stereoscopically presented familiar objects, especially when no visual feedback is available. But what about the influence on vergence movements?

### Familiar size does not influence vergence movements (at least in these experiments)

In both experiments, vergence distances were highly correlated with object distances as specified by both binocular disparity and familiar size (congruent trials). While in experiment 1, computed vergence distance was on average about 3 cm shorter than expected, in experiment 2, vergence distances in congruent trials corresponded quite precisely to the expected distances. Thus, the deviations in experiment 1 were probably due to the difference in luminance between the calibration screen (small grey ring on black background) and the stimulus screen (larger colored object on black background).

When familiar size-specified distance was in conflict with disparity-specified distance, vergence distance followed the distance as specified by binocular disparity. The effect of familiar size-specified distance on vergence distance was significant in experiment 1, but could not be replicated in experiment 2. This was despite the sufficiently large power of experiment 2. The two experiments differed only in luminance conditions. It is therefore likely, that the effect of familiar size-specified distance on vergence distance found in experiment 1 was due to the pupil size artefact described by Hooge and colleagues [76]. Thus, taken together our results suggest that the familiar size of a stimulus is not taken into account when executing a vergence movement towards it.

Vergence latency, the time from object onset until the vergence movement started, was neither influenced by binocular disparity nor by familiar size in experiment 1. In experiment 2, there was a significant effect of familiar size on vergence latency. However, the effect explained less than 1% of the variance and the mean difference between a familiar-size specified distance of 30 cm compared to 40 cm was only 2 ms. Thus, taken together the results suggest that vergence latency is not substantially influenced by familiar size. This is opposed to the results of Grosjean and colleagues [35], who found longer latencies for conflict trials. However, in their setting, binocular disparity suggested a divergence movement whereas the hollow mask illusion suggested a convergence movement. The authors interpreted the delay as the time it took the oculomotor system to override the divergent response based on binocular disparity. In our setting, both binocular disparity and familiar size suggested a convergence movement; there was no conflict concerning the direction but only concerning the amplitude of the vergence movement. Thus, there was no conflicting response that needed time to be inhibited.

Maximal vergence velocity depended on binocular disparity with larger velocities for smaller object distances in both experiments. This result was expected as vergence movements with larger amplitudes are known to have higher maximal velocities [24]. Maximal vergence velocity was not significantly influenced by the manipulation of familiar size in experiment 1. In experiment 2, the effect of familiar size-specified distance on maximal vergence velocity was significant. When familiar size specified a smaller object distance and thus a larger amplitude of the movement, maximal vergence velocity was slightly higher. However, the effect explained less than 1% of the variance. Thus, taken together the results suggest that maximal vergence velocity is not substantially influenced by familiar size.To sum up, we analyzed three parameters describing vergence movements: Vergence distance, vergence latency, and maximal vergence velocity. Taken together, the results of the two experiments suggest that all three parameters are largely unaffected by familiar size. Thus, familiar size does not seem to influence vergence movements in this experimental setting in a substantial manner. Instead, vergence movements closely followed the distance specified by binocular disparity. This is in line with models on the control of vergence movements [23, 24] and experimental studies showing that vergence movements are based on binocular disparity [30–32].

There are several limitations of the current experiment, which restrict the generalization of the results and pose questions for further research:

### Limitations and open questions

We used virtual instead of real objects in order to be able to present objects in several sizes and several distances with high precision, and to have a high level of experimental control. However, this raises the question whether the results can be generalized to reaching and vergence movements towards real objects. There are only few studies on reaching or grasping presenting both virtual and real objects. In general, the results for virtual and real objects are similar: manipulations had the same effect, although the movements were in part less precise towards virtual objects and the magnitude of the effects could differ [79, 88, 89]. It could be that the effect of familiar size on reaching movements is overestimated by our experiment: Presenting a single virtual object has to be classified as a reduced cue condition and it is known that the influence of familiar size on depth perception is stronger in reduced cue conditions compared to full-cue environments [90]. Further, reaching towards a virtual object without haptic feedback is a rather artificial action. In their review, Milner and Goodale [6] suggest that only skilled actions with the dominant hand towards visible targets are fully under the control of the dorsal stream. One could assume that information from the ventral stream including depth information from familiar size was used for the reaching movement in our experiment because the action was not a highly skilled action. We therefore conclude that the information from familiar size is used for the control of reaching movement towards virtual objects and presumably also towards real objects, but probably to a smaller extent.

All studies, in which familiar objects are repeatedly presented, have the limitation that the method does not allow us to differentiate between the effect of long-term familiarity with stored sizes in long-term memory as opposed to new associations learned from the congruent trials. This means that participants might have learned how big the tennis ball was in most of the trials instead of referring to their knowledge about tennis balls. Indeed, experiments with a priori unknown objects have shown that such associations can be acquired and used for programming grasping movements when feedback is available, objects are fully textured, and only few distances are used [10–15]. In contrast to these conditions, we presented six different objects at five different distances, which should have minimized the ability to learn object-size associations. However, we cannot rule out the possibility that part of the effect of familiar size on reaching movements is not due to long-term but due to short-term familiarity.

In our experiments, participants differed in the weight they assigned to each depth cue for the control of their reaching movement. While some gave more weight to binocular disparity, others oriented themselves mostly on familiar size. The reasons for this different weighting are still an open research question.

We did not find an effect of familiar size on vergence movements. This does, of course, not rule out the possibility that there is an effect under differing experimental conditions. At least since the work of Yarbus [91], it is known that eye movements are influenced by instruction. Here, vergence movements were measured in an experiment on distance perception. Participants fixated the objects to determine their distance in order to be able to reach towards them. It is unclear whether the results for vergence movements might be different with a different instruction. This could be tested in a replication using, for example, a free-viewing or a memorization paradigm. Further, vergence movements can be divided into a transient and a sustained part [92]. The transient part is fast and preprogrammed and initiates the response, while the sustained part is slow and under feedback control and brings the eyes to the final, accurate position [92, 93]. It could be possible that there is an effect of familiar size on vergence distance when the vergence movement is first programmed (in the transient part), but is corrected during the movement by the sustained part through the disparity feedback loop. In our data, corrective movements were not clearly visible in aggregated or raw data. However, because vergence movements are slow compared to other eye movements, preprogrammed vergence distance might be gradually corrected during the movement. The effect of familiar size on preprogrammed vergence distance could be tested in a replication of the experiment, in which the objects are blanked out when the vergence movement starts.

## Conclusions

Using stereoscopic images of everyday familiar objects we replicated the result that familiar size is taken into account for programming and executing reaching movements towards familiar objects, especially when no visual feedback is available. On the other hand, vergence movements were largely unaffected by familiar size in the same setting and closely followed binocular disparity. This novel finding emphasizes differences in mechanisms of eye movement control and those of manual actions such as reaching and grasping. Since this is the first study that explores the effect of familiar size on vergence movements, many questions concerning neurophysiological mechanisms behind these differences are open for future investigations.

## Supporting information

S1 Table Final linear mixed models for experiment 1 and experiment 2 (using the formula notation of the R package lme4). (DOCX)
