# Supplementary material for: Size matters: How reaching and vergence movements are influenced by the familiar size of stereoscopically presented objects
Source: PLoS One. 2019 Nov 20;14(11):e0225311. doi: 10.1371/journal.pone.0225311 (PMC6867642; doi:10.1371/journal.pone.0225311)
Supplement: S1 Table — (DOCX) [file pone.0225311.s001.docx]

**S1 Table. Final linear mixed models for experiment 1 and experiment 2 (using the formula notation of the R package lme4).**

| **Experiment 1** | **Experiment 2** |
| --- | --- |
| Vergence latency ~  Disparity-specified distance * Familiar size-specified distance +  (1 \| Participant) +  (1 \| Participant:Object) | Vergence latency ~  Disparity-specified distance * Familiar size-specified distance +  (Familiar size-specified distance \|\| Participant) +  (1 \| Object) +  (0 + Disparity-specified distance \| Participant:Object) |
| Maximal vergence velocity ~  Disparity-specified distance * Familiar size-specified distance +  (1 \| Participant) +  (Familiar size-specified distance \|\| Participant:Object) | Maximal vergence velocity ~  Disparity-specified distance * Familiar size-specified distance +  (Disparity-specified distance \|\| Participant) +  (1 \| Object) +  (1 \| Participant:Object) |
| Vergence distance ~  Disparity-specified distance * Familiar size-specified distance +  (Disparity-specified distance \|\| Participant) +  (1 \| Object) | Vergence distance ~  Disparity-specified distance * Familiar size-specified distance +  (Disparity-specified distance \| Participant) +  (1 \| Object) +  (Disparity-specified distance \|\| Participant:Object) |
| Reaching distance ~  Reaching type * Disparity-specified distance * Familiar size-specified distance +  (Reaching type * (Disparity-specified distance + Familiar size-specified distance) \|\|  Participant) +  (1 \| Object) +  ((Reaching type + Disparity-specified distance + Familiar size-specified distance)^2 - Disparity-specified distance \|\| Participant:Object) | Reaching distance ~  Reaching type * Disparity-specified distance * Familiar size-specified distance +  (Reaching type * Disparity-specified distance * Familiar size-specified distance -  Disparity-specified distance:Familiar size-specified distance \|\| Participant) +  (Disparity-specified distance \| Object) +  ((Reaching type + Disparity-specified distance + Familiar size-specified distance)^2 -  Disparity-specified distance \|\| Participant:Object) |
| Reaching duration ~  Reaching type * Trial type +  (Reaching type * Trial type – Trial type \| Participant) +  (0 + Reaching type \| Object) +  (1 \| Participant:Object) | Reaching duration ~  Reaching type * Trial type +  (Reaching type \| Participant) +  (0 + Reaching type:Trial type \| Participant:Object) |
